# The association between C-reactive protein-triglyceride glucose index and all-cause mortality in patients with cardiovascular-kidney-metabolic syndrome: a single-center retrospective cohort study

**DOI:** 10.3389/fcvm.2026.1832873

**Published:** 2026-07-08

**Authors:** Jiacai Lin, Shaobin Qiu, Shuling Su, Weixin Ni, Yongluan Lin, Xiaobin Ni

**Affiliations:** 1 Department of Cardiology, The First Affiliated Hospital of Shantou University Medical College, Shantou, Guangdong, China; 2 Shantou University Medical College, Shantou, Guangdong, China; 3 Key Laboratory for Prevention and Control of Arrhythmia and Panvascular Disease, Shantou, Guangdong, China

**Keywords:** all-cause mortality, cardiovascular-kidney-metabolic syndrome, coronary heart disease, C-reactive protein-triglyceride glucose index, triglyceride glucose Index

## Abstract

**Background:**

Patients with cardiovascular-kidney-metabolic (CKM) syndrome have high mortality risk. The C-reactive protein–triglyceride glucose index (CTI) integrates inflammation and insulin resistance, yet its stage-specific prognostic value is undefined.

**Methods and results:**

This retrospective cohort study included 8,632 CKM patients (2020–2025), stratified by CTI quartiles. All-cause mortality was assessed using adjusted Cox models. Among 860 deaths (10.0%), CTI was an independent mortality predictor in both stage 0–3 (HR 1.68, 95% CI 1.30–2.18) and stage 4 CKM (HR 1.54, 1.33–1.77). Compared with Q1, Q4 patients had significantly higher risk (stage 0–3: HR 4.57, 2.32–8.99; stage 4: HR 2.26, 1.64–3.13). A linear dose-response relationship was confirmed (*P* for nonlinear >0.05). CTI showed superior 1-year predictive performance over TyG index (AUC: stage 0–3, 0.73 vs. 0.62; stage 4, 0.69 vs. 0.51).

**Conclusion:**

CTI is a potent, independent predictor of all-cause mortality in CKM, with a linear dose-response relationship. By integrating inflammatory and metabolic pathways, it provides superior risk stratification over TyG index, highlighting its clinical utility.

## Introduction

Cardiovascular-kidney-metabolic syndrome (CKM) is a complex clinical condition characterized by the interplay and coexistence of cardiovascular disease, chronic kidney disease, and metabolic disorders. Patients with CKM face a poor prognosis and high all-cause mortality (1, 2). As the global burden of metabolic diseases continues to rise, the prevalence of CKM is increasing, posing a significant public health challenge. Consequently, there is a pressing clinical need for novel biomarkers capable of effectively stratifying risk in CKM patients, enabling the early identification of high-risk individuals for tailored interventions to improve outcomes.

Inflammation and insulin resistance (IR) are widely recognized as two core pathophysiological mechanisms driving the development and progression of CKM (3, 4). However, conventional biomarkers often assess these pathways in isolation. For instance, C-reactive protein (CRP) primarily reflects systemic inflammation (5), while the triglyceride-glucose (TyG) index is a surrogate marker of IR (6). Given that the pathogenesis of CKM involves complex interactions across multiple systems and pathways, a unidimensional assessment fails to capture the overall pathophysiological burden of the syndrome.

The C-reactive protein-triglyceride glucose index (CTI) is an emerging composite biomarker calculated as the product of CRP and the TyG index. By simultaneously integrating the two critical pathways of systemic inflammation and metabolic dysregulation, CTI theoretically offers a more comprehensive assessment of the pathological burden in CKM patients (7, 8). Recent studies have confirmed that CTI is associated with adverse outcomes in individual diseases such as coronary heart disease and heart failure (7, 9). Nonetheless, its prognostic value within the broader CKM spectrum, and particularly whether its predictive performance differs across stages of CKM severity (e.g., stage 0–3 vs. stage 4), remains unclear. Furthermore, evidence is lacking to confirm that the association between CTI and mortality is independent of traditional risk factors.

Therefore, this study aimed to investigate the association between CTI and all-cause mortality in patients with CKM using a large retrospective cohort from a single center, with a particular focus on its predictive value across CKM stages. Elucidating the prognostic significance of CTI could not only deepen the understanding of the central role of the “inflammation-metabolism axis” in CKM but also potentially provide a novel, simple, and effective tool for risk stratification in clinical practice.

## Methods

### Study design and population

This retrospective cohort study utilized data from the Eastern Guangdong Health and Disease Cohort Platform (EGHDCP). We analyzed a sub-cohort of patients with Coronary Heart Disease (CHD) from this platform, encompassing participants enrolled between January 1, 2020, and February 28, 2025.

**Figure 1:**
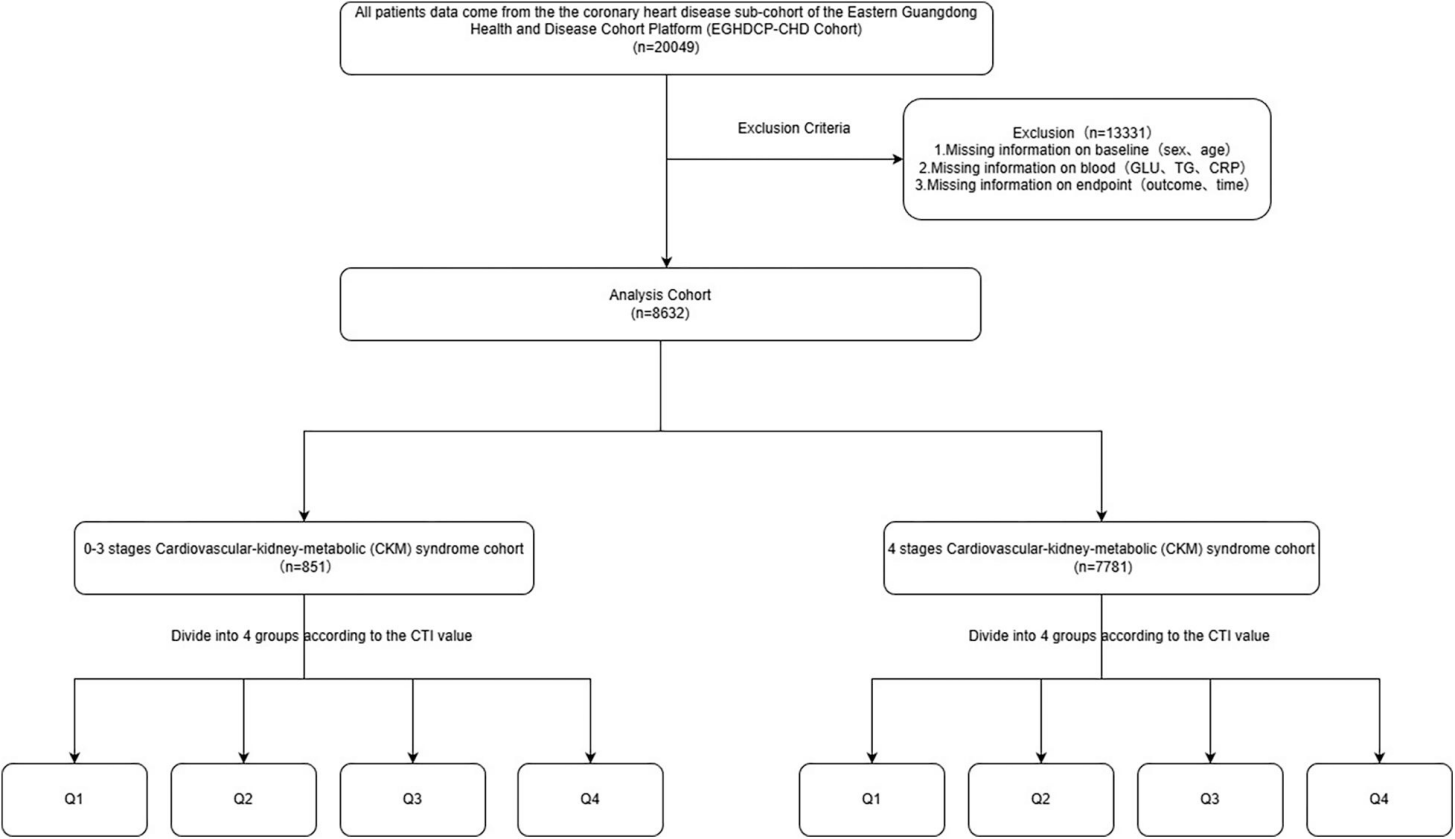
The flow chart of participant selection.

Adults (age ≥ 18 years) with a definitive diagnosis of Cardiovascular-Kidney-Metabolic Syndrome (CKM), staged according to the American Heart Association (AHA) criteria (1), were eligible for inclusion. The key exclusion criteria were: (1) missing baseline data essential for calculating the C-reactive protein-triglyceride glucose index (CTI), namely C-reactive protein (CRP), triglyceride (TG), or fasting blood glucose (GLU) values; and (2) a follow-up duration of less than 1 day. A total of 8,632 patients who met the criteria constituted the final study population. A flow diagram of the participant selection process is provided in Figure 1.

Baseline clinical data including demographic characteristics, coexisting conditions and laboratory examination were collected. All data were extracted from the clinical electronic management system of the EGHDCP database. The EGHDCP cohort research was approved by the Medical Ethics Committee of the First Affiliated Hospital of Shantou University Medical College (B-2025-065).

### CTI measurement

The primary exposure, CTI, was calculated using the following formula (10): CTI = 0.412 × Ln (CRP [mg/L]) + Ln (TG [mg/dL] × GLU [mg/dL])/2. The formula incorporates natural logarithmic transformations (ln) to normalize the skewed distribution of CRP and TyG values. The coefficient 0.412 for ln(CRP) is derived from previous validation studies [e.g., Ruan et al., (10), Front Endocrinol] to balance the weighted contribution of inflammation relative to metabolic dysregulation, ensuring the composite index reflects the synergistic burden of both pathways. For analysis, CTI was treated both as a continuous variable and as a categorical variable by dividing patients into quartiles (Q1–Q4).

### Endpoint and definition

CHD in EGHDCP-CHD cohort was identified by ICD-10 codes (I20-I25), The AHA Presidential Advisory Statement (1) lists the stages of CKM syndrome as follows: Stage 0: Absence of CKM risk factors. Stage 1: overweight or dysfunctional adiposity. Stage 2: Presence of metabolic disorders,including hypertension, diabetes and elevated triglycerides, or CKD. Stage 3: Subclinical CVD in the context of CKM syndrome. Stage 4:Clinical CVD in CKM. Supplementary File S1 details the concrete stage criteria for CKM syndrome.

The endpoint of the study was defined as all-cause mortality. In EGHDCP-CHD cohort, if patients died in the period of hospitalization, the time of death was recorded. Conversely, if no patient died during hospitalization, we routinely collected survival status and date of death from all participants via telephone follow-up. The time of the endpoint was defined as the time of death, the time of missing follow-up or the last follow-up time, which depended on the event which firstly occurred among them.

### Covariates

We adjusted for a comprehensive set of potential confounders selected based on clinical knowledge and prior literature. These covariates included demographics (e.g., age, sex), vital signs (systolic and diastolic blood pressure), key comorbidities (hypertension, diabetes mellitus, renal dysfunction), and a wide range of laboratory parameters (including measures of inflammation, metabolism, cardiac, renal, and hepatic function). A complete list of all covariates is presented in Supplementary File S2 alongside the baseline characteristics of the study population.

### Statistical analysis

Baseline characteristics of the study participants are presented as means ± standard deviations or medians with interquartile ranges for continuous variables, and as numbers with percentages for categorical variables. Differences across CTI quartiles were compared using one-way ANOVA, Kruskal–Wallis tests, or Chi-Square tests, as appropriate.

The association between CTI and all-cause mortality was evaluated using Cox proportional hazards regression models. We constructed three sequentially adjusted models to assess the robustness of this association: The Crude Model was unadjusted. Model 1 was adjusted for sex and age. Model 2 (Fully Adjusted Model) was further adjusted for a comprehensive set of potential confounders, including demographics, vital signs, key comorbidities, and a wide range of laboratory parameters. The complete list of covariates adjusted for in Model 2 is presented in Supplementary File S2 alongside the baseline characteristics. Hazard ratio (HR) with their corresponding 95% confidence interval (CI) were calculated for both CTI as a continuous variable and as quartile. The linear trend across quartile was tested by treating the median value of each quartile as a continuous variable in the models. The proportional hazards assumption was verified using Schoenfeld residuals. The dose-response relationship between CTI as a continuous variable and mortality risk was flexibly modeled and visualized using restricted cubic splines (RCS) with 4 knots. Subgroup analyses were performed by stratifying the cohort based on clinically relevant factors, and interaction terms were incorporated into the Cox models to test for effect modification. To evaluate and compare the predictive performance of the C-reactive protein-triglyceride glucose index (CTI, Model 1) and the triglyceride glucose index (TyG, Model 2) for all-cause mortality, we performed a time-dependent ROC analysis. The analysis was stratified by CKM severity stage (Stage 0–3 and Stage 4). The area under the curve (AUC) and the concordance index (C-index) were used as the primary metrics to assess model discrimination. The primary evaluation time point was set at 1 year, with a secondary analysis at 30 days to examine short-term predictive ability. Time-dependent ROC curves and C-index curves were plotted to dynamically visualize the discriminatory performance of both indices throughout the entire follow-up period.

All statistical analyses were conducted using R software (version 4.3.0) and the Fengrui Statistical Software (version 2.2.0). *p* < 0.05 was considered as statistically significant.

## Results

### Study population and baseline characteristics

A total of 8,632 patients with CKM were included in the final analysis. The baseline characteristics of the entire study population and stratified by CTI quartiles are summarized in Supplementary File S2. The study cohort had a mean age of 67.2 ± 11.4 years, and 67.3% were male. Patients in the highest CTI quartile (Q4) exhibited a more severe clinical profile compared to those in the lowest quartile (Q1). Specifically, the Q4 group had significantly higher levels of inflammatory and metabolic markers (e.g., CRP, TG, GLU, HbA1c), cardiac stress markers (NT-proBNP), and indicators of renal dysfunction (Creatinine). They also had a higher prevalence of comorbidities such as hypertension, diabetes mellitus, and renal dysfunction, and were more frequently treated with medications including diuretics, amiodarone, and digoxin (all *P* for trend <0.001). Over a median follow-up period, 860 (10.0%) all-cause death events were recorded, with a clear increasing trend in mortality rates from the lowest to the highest CTI quartile (Q1: 3.3%; Q2: 6.7%; Q3: 10.3%; Q4: 19.5%; *P* < 0.001).

### Association between CTI and All-cause mortality

The associations of CTI with all-cause mortality, stratified by CKM stages (0–3 vs. 4), are presented in Table 1. In patients with stage 0–3 CKM, a higher CTI was strongly associated with an increased risk of all-cause mortality. In the fully adjusted model (Model 2), each unit increase in CTI (as a continuous variable) was associated with a 68% increased risk of death (HR 1.68, 95% CI 1.30–2.18, *P* = 0.001). When analyzed by quartiles, a significant dose-response relationship was observed (*P* for trend <0.001). Compared to patients in Q1, the risk of mortality was significantly higher in Q2 (HR 1.93, 95% CI 1.00–3.74, *P* = 0.052), Q3 (HR 3.03, 95% CI 1.59–5.77, *P* = 0.001), and Q4 (HR 4.57, 95% CI 2.32–8.99, *P* <0.001). In patients with stage 4 CKM, CTI remained a significant predictor of mortality after full adjustment. The hazard ratio per unit increase in CTI was 1.54 (95% CI 1.33–1.77, *P* < 0.001). Similarly, a graded increase in mortality risk was observed across CTI quartiles (*P* for trend < 0.001), with patients in Q4 having a 126% higher risk compared to those in Q1 (HR 2.26, 95% CI 1.64–3.13, *P* < 0.001).

**Table 1:** Cox regression models for CTI and all-cause mortality.

CTI	Crude model^a^		Model 1^b^		Model 2^c^	
	95% CI	*P*	95% CI	*P*	95% CI	*P*
Stage 0–3 CKM						
Continuous	2.5 (2.08–3)	<0.001	2.41 (2–2.9)	<0.001	1.68 (1.3–2.18)	0.001
Categories						
Q1	1(Ref)		1(Ref)		1(Ref)	
Q2	2.17 (1.17–4.03)	0.015	2.1 (1.13–3.91)	0.019	1.93 (1–3.74)	0.052
Q3	4.25 (2.37–7.62)	<0.001	4.04 (2.25–7.26)	<0.001	3.03 (1.59–5.77)	0.001
Q4	8.47 (4.8–14.93)	<0.001	8.08 (4.58–14.26)	<0.001	4.57 (2.32–8.99)	<0.001
*P* for trend	2.01 (1.73–2.33)	<0.001	1.99 (1.71–2.31)	<0.001	1.61 (1.33–1.94)	<0.001
Stage 4 CKM						
Continuous	2.4 (2.15–2.68)	<0.001	2.49 (2.22–2.79)	<0.001	1.54 (1.33–1.77)	<0.001
Categories						
Q1	1(Ref)		1(Ref)		1(Ref)	
Q2	1.7 (1.24–2.33)	0.001	1.63 (1.19–2.24)	0.002	1.46 (1.05–2.03)	0.023
Q3	2.5 (1.85–3.37)	<0.001	2.38 (1.77–3.21)	<0.001	1.75 (1.27–2.41)	0.001
Q4	4.83 (3.64–6.4)	<0.001	4.68 (3.53–6.21)	<0.001	2.26 (1.64–3.13)	<0.001
*P* for trend	1.69 (1.57–1.83)	<0.001	1.69 (1.56–1.83)	<0.001	1.28 (1.17–1.41)	<0.001

CKM, cardiovascular-kidney-metabolic; CTI, C-reactive protein-triglyceride glucose index; HR, Hazard Ratio; CI, Confidence Interval.

Units of CTI: Since CTI is derived from the sum of natural logarithms [ln(mg/L) and ln(mg/dL)], it is a dimensionless continuous index.

a Crude Model: Unadjusted.

b Model 1: Adjusted for Sex and Age.

c Model 2: Adjusted for Sex, Age, Hypertension, Diabetes, Renal dysfunction, DBP, SBP, NT-proBNP, Na, K, CR, TC, HDL-C, LDL-C, AST, ALT, ALB, WBC.

### Restricted cubic spline models

**Figure 2:**
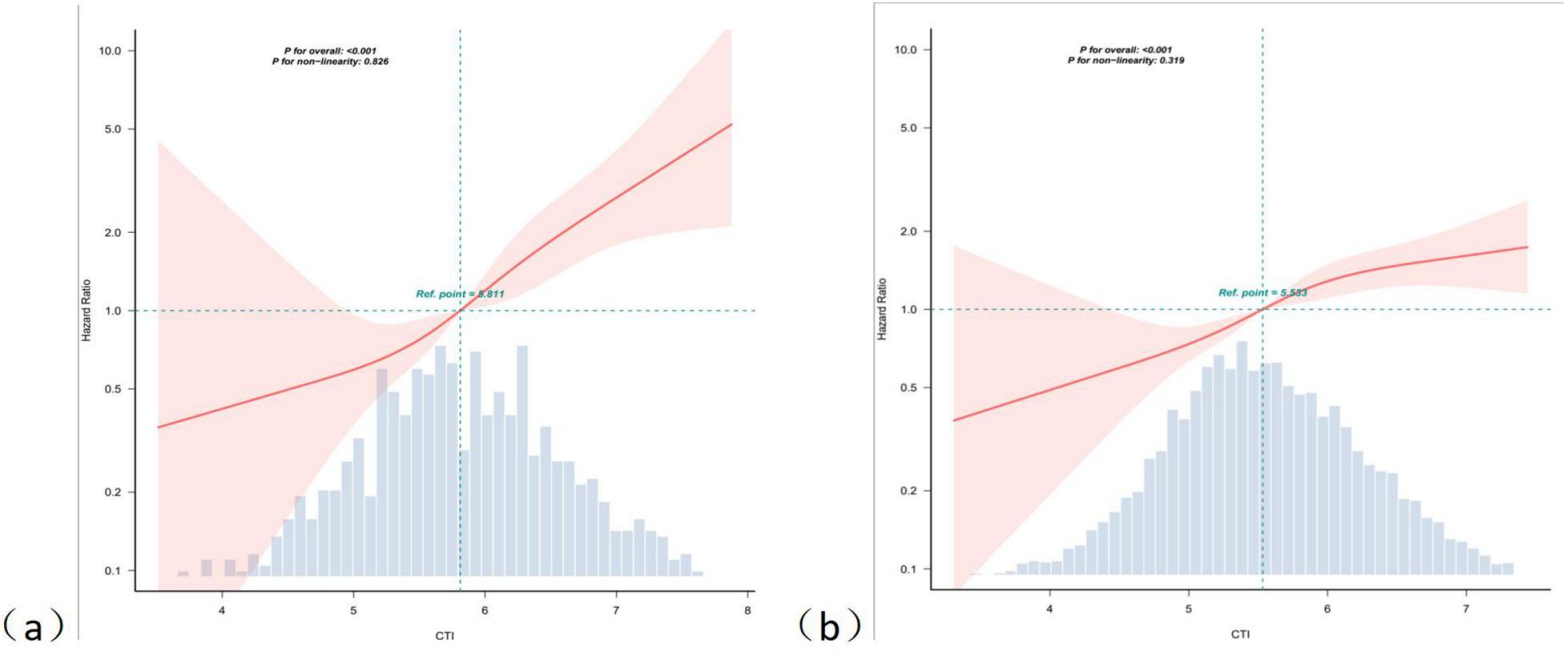
Association between CTI and all-cause mortality of CKM by restricted cubic spline regression. **(a)** Association in the stage 0–3 CKM cohort; **(b)** Association in the stage 4 CKM cohort.

To elucidate the shape of the association between CTI and all-cause mortality beyond simple quartile comparisons, we performed a restricted cubic spline (RCS) analysis. As illustrated in Figure 2, a clear, progressive increase in the log hazard ratio for mortality was observed with rising CTI levels in both the stage 0–3 and stage 4 CKM cohorts.

The analysis revealed a striking pattern: the relationship between CTI and mortality risk was monotonically increasing and largely linear across the majority of the observed CTI range. There was no evidence of a threshold effect or a plateau at higher CTI values within the study population. The likelihood ratio tests for nonlinearity were statistically non-significant (*P* for nonlinearity >0.05 for both CKM stage groups), confirming that the linear model provided an adequate fit to the data.

This continuous, linear dose-response relationship strengthens the causal inference, suggesting that not only is a higher CTI categorically associated with greater risk, but each incremental increase in CTI corresponds to a steady rise in mortality risk. This finding reinforces CTI as a robust gradient measure of pathological burden in CKM patients.

### Survival analysis according to CTI quartiles

**Figure 3:**
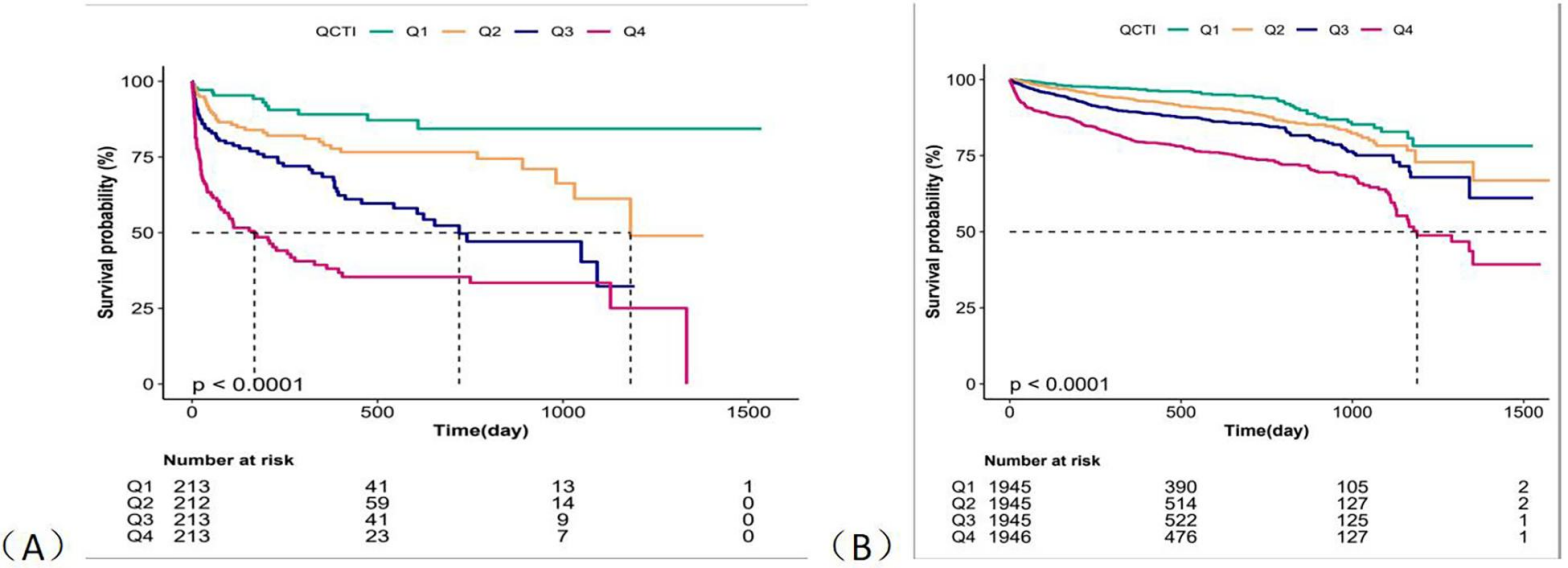
Association between CTI and patient survival by Kaplan–Meier survival curves. **(A)** Association in the stage 0–3 CKM cohort; **(B)** Association in the stage 4 CKM cohort.

The association between CTI levels and all-cause mortality was visually assessed using Kaplan–Meier survival curves, with statistical significance evaluated by the log-rank test. Figure 3 depicts the survival probability over time stratified by CTI quartiles (Q1–4) for the entire cohort.

A striking and statistically significant disparity in survival rates was observed across the CTI quartiles. Patients in the lowest CTI quartile (Q1) demonstrated the most favorable survival probability throughout the follow-up period. In contrast, survival rates progressively worsened with each increasing CTI quartile. The curve for the highest quartile (Q4) consistently displayed the poorest survival outcome, separating from the other curves early in the follow-up and maintaining a substantial gap over time.

The log-rank test for the overall difference among the four survival curves was highly significant (*P* < 0.0001), providing strong evidence against the null hypothesis of no difference in survival distributions between the CTI groups. This clear, graded inverse relationship between baseline CTI level and cumulative survival probability provides compelling graphical evidence of the prognostic value of CTI for risk stratification in CKM patients.

### Subgroup analyses

**Figure 4:**
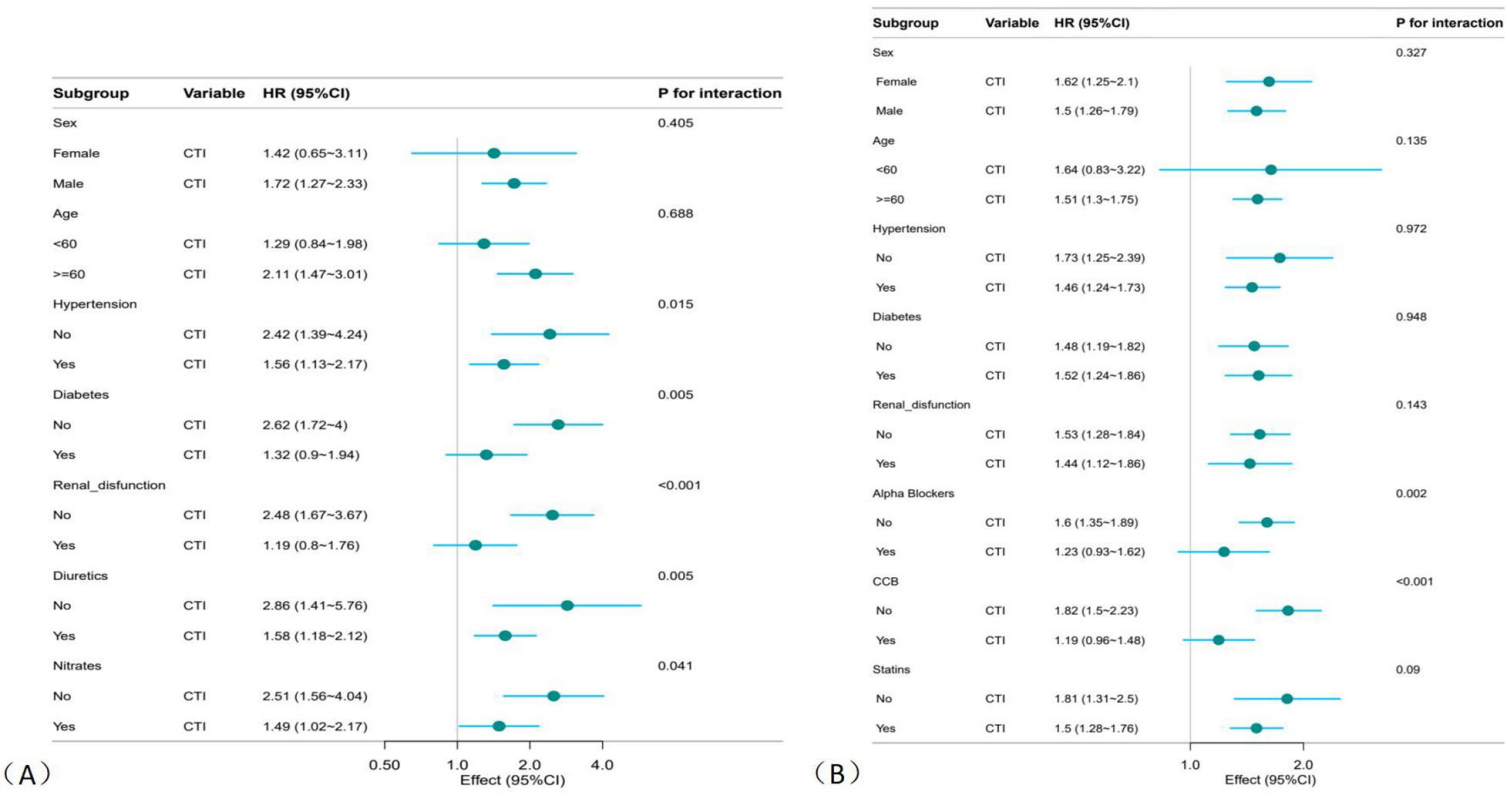
Forest plot of CTI for all-cause mortality in patients with CKM syndrome. **(A)** Forest plot for patients with stage 0–3 CKM syndrome; **(B)** Forest plot for patients with stage 4 CKM syndrome.

Subgroup analyses were performed to assess the consistency of the association between CTI (as a continuous variable) and all-cause mortality across various patient characteristics. The results are presented in Figure 4.

In the stage 0–3 CKM cohort, a higher CTI was associated with an increased risk of all-cause mortality in most patient subgroups. Statistically significant effect modification was observed for several clinical factors, as indicated by interaction *P*-values <0.05. These included hypertension (*P* for interaction = 0.015), diabetes (*P* for interaction = 0.005), renal dysfunction (*P* for interaction < 0.001), use of diuretics (*P* for interaction = 0.005), and use of nitrates (*P* for interaction = 0.041).

Notably, the magnitude of the association between CTI and mortality varied meaningfully across these strata. The hazard was substantially greater among patients without hypertension (adjusted HR 2.42, 95% CI 1.39–4.24) compared to those with hypertension (adj. HR 1.56, 95% CI 1.13–2.17). A similar pattern was evident for the absence of diabetes (adj. HR 2.62, 95% CI 1.72–4.00 vs. 1.32, 95% CI 0.90–1.94 with diabetes), absence of renal dysfunction (adj. HR 2.48, 95% CI 1.67–3.67 vs. 1.19, 95% CI 0.80–1.76 with renal dysfunction), and non-use of diuretics (adj. HR 2.86, 95% CI 1.41–5.76 vs. 1.58, 95% CI 1.18–2.12 with diuretic use). Similarly, the association was stronger among non-users of nitrates (adj. HR 2.51, 95% CI 1.56–4.04) compared to users (adj. HR 1.49, 95% CI 1.02–2.17). No significant interaction was detected for sex or age (*P* for interaction > 0.05).

In the stage 4 CKM cohort, the positive association between CTI and all-cause mortality was also evident across most subgroups. Significant effect modification was observed for the use of specific antihypertensive medication classes. A significant interaction was present for alpha-blockers (*P* for interaction = 0.002), with a significant association in non-users (adj. HR 1.60, 95% CI 1.35–1.89; *P* < 0.001) but not in users (adj. HR 1.23, 95% CI 0.93–1.62; *P* = 0.149). A highly significant interaction was found for calcium channel blockers (CCB) (*P* for interaction <0.001), with a strong association in non-users (adj. HR 1.82, 95% CI 1.50–2.23; *P* < 0.001) that was markedly attenuated and non-significant in users (adj. HR 1.19, 95% CI 0.96–1.48; *P* = 0.114). For statin use, the interaction term approached but did not reach statistical significance (*P* for interaction = 0.09). The point estimates suggested a potentially stronger association in non-users (adj. HR 1.81, 95% CI 1.31–2.50) than in users (adj. HR 1.50, 95% CI 1.28–1.76), though both were significant.

In contrast, no significant interaction was detected for sex, age, hypertension, diabetes, or the other medication classes analyzed in this cohort (all *P* for interaction >0.05), indicating a consistent predictive value of CTI within these subgroups. Supplementary File S3 details the association between CTI and all-cause mortality in CKM syndrome by subgroup analyses.

### Time-dependent receiver operating characteristic (ROC) analysis

This study evaluated the predictive performance of the C-Reactive Protein-Triglyceride Glucose Index (CTI, Model 1) and the Triglyceride Glucose Index (TyG, Model 2) for all-cause mortality in patients with Cardiovascular-Kidney-Metabolic (CKM) Syndrome. The analysis was stratified by disease severity (Stage 0–3 and Stage 4), and model performance was assessed using time-dependent Receiver Operating Characteristic (ROC) analysis, with the Area Under the Curve (AUC) and the concordance index (C-index) as primary metrics.

**Figure 5:**
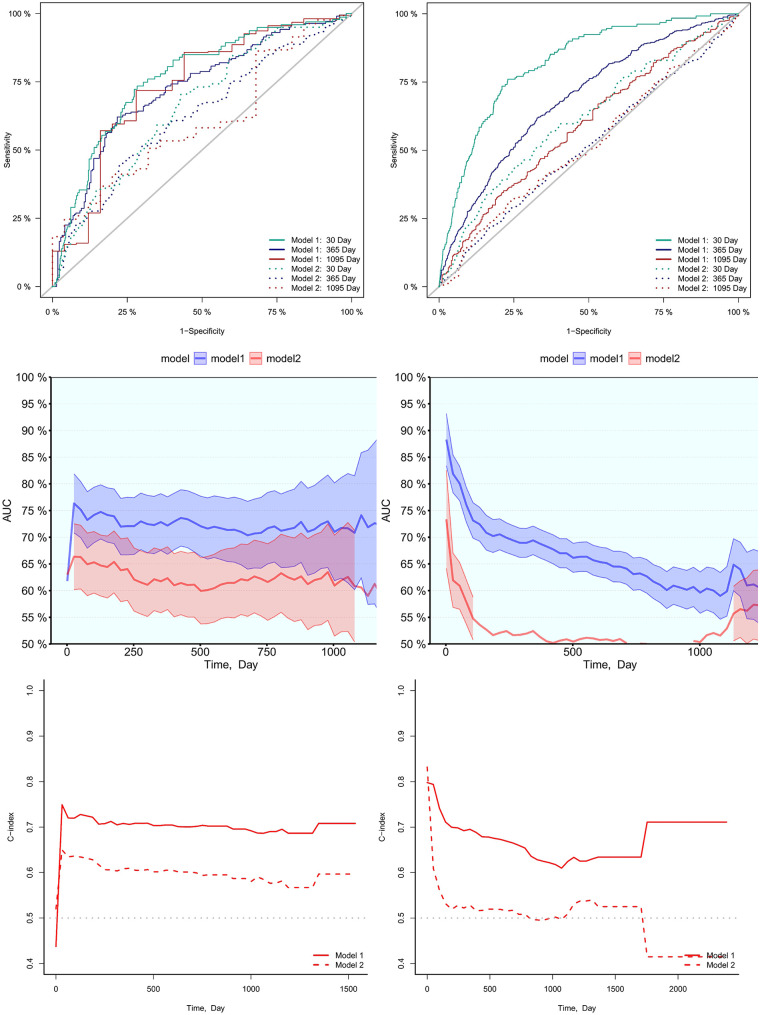
Time-dependent ROC analysis of CTI for all-cause mortality in patients with CKM syndrome. The left column shows the analysis for Stage 0–3 CKM patients, and the right column shows the analysis for Stage 4 CKM patients. Model 1: CTI index; Model 2: TyG index.

At the primary time point of one year, the CTI index demonstrated superior predictive ability for all-cause mortality compared to the TyG index across both patient cohorts. The results are presented in Figure 5.

In Stage 0–3 CKM patients, the AUC for CTI was 0.73 (95% CI: 0.67–0.78), which was higher than the AUC of 0.62 (95% CI: 0.57–0.68) for TyG. The C-index analysis, using data from the closest time point to 365 days, confirmed this advantage. The C-index for CTI was 0.71, compared to 0.61 for TyG.

Similarly, in Stage 4 CKM patients, the performance gap was pronounced. The AUC for CTI was 0.69 (95% CI: 0.66–0.71), substantially outperforming TyG, which had an AUC of 0.51 (95% CI: 0.48–0.54). The C-index analysis yielded consistent results, with values of 0.70 for CTI and 0.53 for TyG.

The secondary analysis at 30 days reinforced the findings of the primary analysis, showing excellent short-term predictive power for the CTI index.

For Stage 0–3 patients, the 30-day AUC for CTI was 0.77 (95% CI: 0.71–0.82), substantially exceeding the TyG AUC of 0.66 (95% CI: 0.60–0.72). The corresponding C-index at the nearest time point was 0.75 for CTI vs. 0.65 for TyG.

In the Stage 4 cohort, CTI also maintained a strong advantage at 30 days, with an AUC of 0.82 (95% CI: 0.79–0.85) compared to 0.62 (95% CI: 0.57–0.66) for TyG. The C-index values were 0.79 for CTI and 0.61 for TyG.

The time-dependent ROC and C-index curves revealed that the predictive discrimination of the CTI index remained consistently higher than that of the TyG index throughout the follow-up period in both Stage 0–3 and Stage 4 patients. The performance of the TyG index often approached the null value (AUC = 0.50) at later time points, particularly in the high-risk Stage 4 group, indicating limited long-term prognostic value.

## Discussion

### Synopsis of Key findings

This study demonstrates that the C-reactive protein-triglyceride glucose index (CTI), an integrative biomarker reflecting concomitant inflammation and insulin resistance, serves as a powerful, independent predictor of all-cause mortality in patients with cardiovascular-kidney-metabolic (CKM) syndrome. Four principal findings emerge from our analysis: a strong, graded association between higher CTI levels and increased mortality risk, independent of multivariable adjustments; a continuous, linear dose-response relationship without evidence of a threshold; significant effect modification by CKM stage, with a stronger association in earlier stages; and robust prognostic consistency across nearly all clinical subgroups, highlighting its broad generalizability.

### Incremental clinical value

The time-dependent analyses demonstrated that the prognostic performance of CTI was most pronounced during mid-term follow-up, particularly at 1 year, suggesting that the integrated inflammatory–metabolic burden captured by CTI exerts sustained rather than short-lived prognostic effects in CKM. Notably, the TyG index demonstrated poor discriminative ability in Stage 4 CKM patients (AUC 0.51), performing at chance level. This may be explained by the pathophysiology of advanced CKM (Stage 4), where “metabolic exhaustion” or the dominance of established structural cardiovascular damage and renal dysfunction may render isolated markers of insulin resistance less informative for short-to-mid-term mortality prediction. In contrast, the CTI's incorporation of the CRP component captures the superimposed inflammatory burden, which is often amplified in advanced disease states (e.g., heart failure-related congestion and systemic inflammation), thereby restoring prognostic value where TyG fails.

### Stage-specific implications

The differential strength of association across CKM stages carries important clinical implications. The more pronounced hazard ratios in stage 0–3 patients suggest that the metabolic-inflammator*y* axis measured by CTI may be especially critical in driving initial disease progression. This identifies a potential window of opportunity wherein aggressive targeting of these pathways could alter the clinical trajectory. In contrast, the attenuated—though still significant—association in stage 4 patients implies that in advanced disease, other factors such as irreversible organ failure may dominate the prognostic landscape. This stage-specific prognostic performance could refine risk stratification paradigms, allowing for more personalized management strategies.

### Pathophysiological mechanisms and context with existing evidence

The robust association between CTI and mortality in CKM syndrome is firmly underpinned by a compelling pathophysiological rationale that interlinks chronic inflammation, insulin resistance (IR), and progressive multi-organ dysfunction. Our findings posit that the CTI quantitatively captures the intensity of this core pathological axis. The linear dose-response relationship observed suggests that the maladaptive processes quantified by the CTI exert continuous, cumulative damage, with no safe threshold.

The synergy captured by the CTI originates from a well-established vicious cycle. In CKM syndrome, dysfunctional adipose tissue drives chronic low-grade inflammation, characterized by elevated levels of cytokines such as TNF-α and IL-6, which stimulate hepatic CRP production (10). These inflammatory mediators simultaneously activate intracellular stress pathways (e.g., IKK*β*/NF-*κ*B and JNK) that impair insulin signaling by promoting serine phosphorylation of insulin receptor substrate (IRS) proteins, leading to systemic IR (3, 11). The resulting hyperglycemia and dyslipidemia further exacerbate inflammation through mechanisms involving oxidative stress and inflammasome activation, creating a feed-forward loop of metabolic and inflammatory dysfunction (12, 13).

This inflammation-IR axis inflicts direct damage across CKM-relevant organ systems. It promotes endothelial dysfunction and the development of unstable atherosclerotic plaques in the vasculature (14, 15), while also driving renal fibrosis and podocyte injury, accelerating cardiorenal syndrome (16). The CTI, by integrating measures of both inflammation (CRP) and metabolic dysregulation (TyG index), thus provides a more holistic gauge of this pathophysiological burden than either component alone.

Our findings significantly extend the existing literature on composite biomarkers. While the prognostic value of CRP (17) and the TyG index (4, 5) individually is recognized, research on the combined CTI is emerging. A recent study in critically ill coronary heart disease patients found CTI predicted in-hospital mortality (7), and another in a national cohort linked it to stroke risk in early CKM (8). Our study solidifies this concept within the full CKM spectrum, demonstrating its prognostic power across disease stages and its linear relationship with mortality risk, thereby strengthening the case for its clinical utility.

### Robustness and generalizability of findings

The robustness of our findings is reinforced by their consistency across a wide array of clinically relevant subgroups, including those defined by age, sex, and comorbid conditions. This stability enhances the biomarker's potential for broad application. The observed interactions with specific medication classes, while requiring validation, generate plausible hypotheses about how existing pharmacotherapies might modulate the risk conveyed by a high CTI.

## Study limitations

The current study has several limitations that should be acknowledged. The single-center, retrospective design may limit generalizability, and despite rigorous statistical adjustments, the potential for residual confounding remains. Furthermore, CTI was measured at a single timepoint, which precludes assessment of its dynamic changes and their relationship with outcomes. The findings would also benefit from confirmation in more diverse, multi-ethnic and international populations. Finally, the observational nature of this study means that the reported associations do not establish causality and should be interpreted as such; further mechanistic and interventional research would be valuable to elucidate the underlying relationships.

## Future directions

In conclusion, the CTI emerges as a readily accessible, low-cost prognostic tool that effectively captures the integrated burden of inflammation and metabolic dysregulation in CKM syndrome. Its strong, independent, and graded association with mortality underscores the central pathophysiological role of this axis. Future studies should focus on validating CTI in external cohorts, establishing standardized risk thresholds, and investigating its utility in guiding targeted therapies against inflammation and insulin resistance. The integration of CTI into clinical risk prediction models may enable more precise prognostication and personalized management for patients across the CKM spectrum.

## Conclusions

This study establishes the C-reactive protein-triglyceride glucose index (CTI) as a clinically meaningful predictor with incremental value for all-cause mortality in patients with Cardiovascular-Kidney-Metabolic (CKM) syndrome. We demonstrate a strong, linear dose-response relationship between CTI levels and mortality risk that remains significant after comprehensive adjustment for established risk factors. These findings highlight the central pathophysiological importance of the inflammation-insulin resistance axis in CKM progression.

## Data Availability

The data analyzed in this study is subject to the following licenses/restrictions: Due to policy restrictions, we are unable to provide individual patient data from the First Affiliated Hospital of Shantou University Medical College. Requests to access these datasets should be directed to nixiaobin1983@163.com.
