# Supplementary material for: The association between C-reactive protein-triglyceride glucose index and all-cause mortality in patients with cardiovascular-kidney-metabolic syndrome: a single-center retrospective cohort study
Source: Front Cardiovasc Med. 2026 Jul 8;13:1832873. doi: 10.3389/fcvm.2026.1832873 (PMC13388307; doi:10.3389/fcvm.2026.1832873)
Supplement: Supplementary file 1 [file Supplementaryfile1.docx]

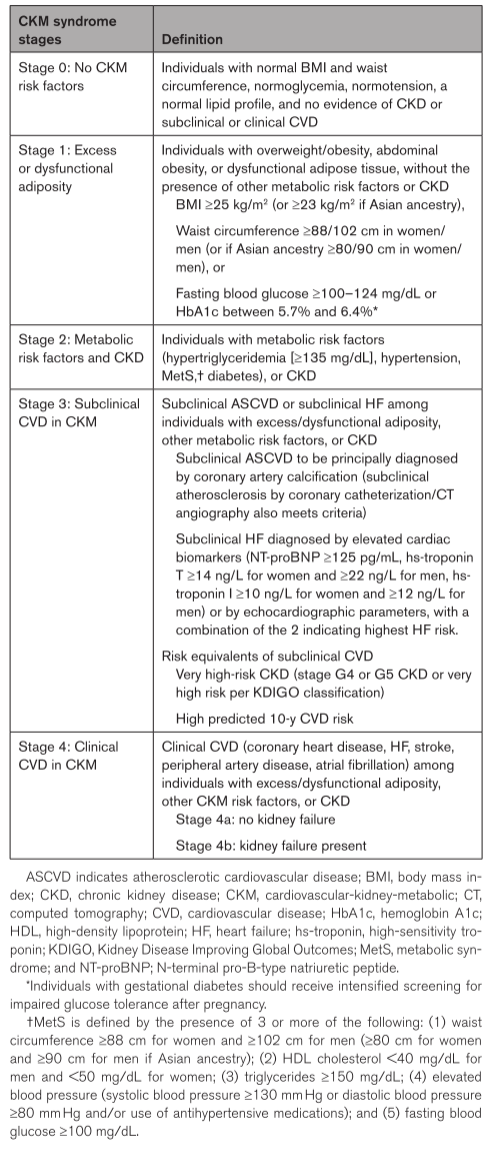
Supplementary File S1：Definitions of CKM syndrome stages.（figure coms from《Cardiovascular-Kidney-Metabolic Health:A Presidential Advisory From the American Heart Association》（DOI：10.1161/CIR.0000000000001184）
