# Supplementary material for: The association between C-reactive protein-triglyceride glucose index and all-cause mortality in patients with cardiovascular-kidney-metabolic syndrome: a single-center retrospective cohort study
Source: Front Cardiovasc Med. 2026 Jul 8;13:1832873. doi: 10.3389/fcvm.2026.1832873 (PMC13388307; doi:10.3389/fcvm.2026.1832873)
Supplement: Supplementary file 2 [file Supplementaryfile2.docx]

Supplementary File：Table S2.Table of Baseline Demographic Characteristics

| Variables | Total (n = 8632) | Q1 (n = 2158) | Q2 (n = 2158) | Q3 (n = 2158) | Q4 (n = 2158) | *p* |
| --- | --- | --- | --- | --- | --- | --- |
| Sex,n(%) |  |  |  |  |  | 0.268 |
| Female | 2819 (32.7) | 671 (31.1) | 731 (33.9) | 711 (32.9) | 706 (32.7) |  |
| Male | 5813 (67.3) | 1487 (68.9) | 1427 (66.1) | 1447 (67.1) | 1452 (67.3) |  |
| Age | 67.2 ± 11.4 | 66.4 ± 10.7 | 67.1 ± 11.3 | 67.5 ± 11.8 | 67.7 ± 11.9 | < 0.001 |
| CRP | 7.6 (3.0, 25.7) | 2.0 (1.2, 3.0) | 5.1 (3.3, 8.1) | 13.4 (7.3, 23.4) | 62.3 (27.6, 102.0) | < 0.001 |
| TG | 1.2 (0.9, 1.7) | 0.9 (0.7, 1.2) | 1.2 (0.9, 1.6) | 1.3 (0.9, 1.9) | 1.5 (1.1, 2.2) | < 0.001 |
| GLU | 6.6 ± 3.0 | 5.4 ± 1.5 | 6.0 ± 2.0 | 6.8 ± 2.8 | 8.4 ± 4.2 | < 0.001 |
| HBA1c | 7.1 ± 1.7 | 6.5 ± 1.1 | 6.9 ± 1.5 | 7.3 ± 1.8 | 7.7 ± 2.1 | < 0.001 |
| NT-proBNP | 482.1 (98.5, 1771.0) | 129.9 (45.7, 601.6) | 328.8 (77.1, 1292.8) | 688.7 (169.0, 1935.5) | 1279.0 (390.5, 4109.0) | < 0.001 |
| Na | 139.9 ± 3.9 | 140.3 ± 2.9 | 140.1 ± 3.2 | 139.7 ± 3.9 | 139.4 ± 5.2 | < 0.001 |
| K | 3.9 ± 0.5 | 3.9 ± 0.4 | 3.9 ± 0.4 | 3.9 ± 0.4 | 4.0 ± 0.6 | < 0.001 |
| CR | 87.1 (72.0, 112.9) | 78.7 (68.9, 94.3) | 84.1 (71.1, 105.1) | 90.5 (73.9, 116.8) | 102.8(76.9, 169.9) | < 0.001 |
| TC | 4.7 ± 1.4 | 4.5 ± 1.2 | 4.7 ± 1.3 | 4.7 ± 1.4 | 4.8 ± 1.6 | < 0.001 |
| HDLC | 1.1 ± 0.3 | 1.2 ± 0.3 | 1.1 ± 0.3 | 1.0 ± 0.3 | 1.0 ± 0.3 | < 0.001 |
| LDLC | 2.9 ± 1.1 | 2.7 ± 1.0 | 2.9 ± 1.0 | 2.9 ± 1.0 | 2.9 ± 1.2 | < 0.001 |
| AST | 23.1 (17.9, 33.8) | 21.0 (17.3, 26.7) | 23.0 (18.0, 32.1) | 23.6 (18.0, 35.4) | 26.8 (18.8, 47.2) | < 0.001 |
| ALT | 22.4 (14.8, 37.4) | 20.1 (14.5, 29.2) | 22.8 (15.5, 37.8) | 23.2 (14.5, 39.7) | 24.9 (14.9, 47.0) | < 0.001 |
| ALB | 36.9 ± 5.3 | 39.3 ± 4.4 | 37.9 ± 4.8 | 36.4 ± 5.0 | 34.0 ± 5.6 | < 0.001 |
| WBC | 8.5 ± 3.8 | 7.2 ± 2.3 | 7.9 ± 2.8 | 8.5 ± 3.1 | 10.4 ± 5.5 | < 0.001 |
| DBP | 82.0 (73.0, 92.0) | 82.0 (74.0, 91.0) | 83.0 (75.0, 93.0) | 82.0 (74.0, 93.0) | 80.0 (71.0, 93.0) | < 0.001 |
| SBP | 137.0 (122.0, 154.0) | 135.0 (123.0, 150.0) | 138.0 (124.0, 154.0) | 138.0 (122.0, 156.0) | 137.0 (120.0, 156.0) | < 0.001 |
| Alpha_Blockers,n(%) |  |  |  |  |  | < 0.001 |
| No | 7319 (84.8) | 1959 (90.8) | 1888 (87.5) | 1812 (84) | 1660 (76.9) |  |
| Yes | 1313 (15.2) | 199 (9.2) | 270 (12.5) | 346 (16) | 498 (23.1) |  |
| Hypertension,n(%) |  |  |  |  |  | < 0.001 |
| No | 2714 (31.4) | 847 (39.2) | 688 (31.9) | 613 (28.4) | 566 (26.2) |  |
| YES | 5918 (68.6) | 1311 (60.8) | 1470 (68.1) | 1545 (71.6) | 1592 (73.8) |  |
| Diabetes,n(%) |  |  |  |  |  | < 0.001 |
| No | 5109 (59.2) | 1617 (74.9) | 1362 (63.1) | 1140 (52.8) | 990 (45.9) |  |
| Yes | 3523 (40.8) | 541 (25.1) | 796 (36.9) | 1018 (47.2) | 1168 (54.1) |  |
| Coronary_Atherosclerosis,n(%) |  |  |  |  |  | < 0.001 |
| No | 851 ( 9.9) | 131 (6.1) | 165 (7.6) | 211 (9.8) | 344 (15.9) |  |
| Yes | 7781 (90.1) | 2027 (93.9) | 1993 (92.4) | 1947 (90.2) | 1814 (84.1) |  |
| Renal_disfunction,n(%) |  |  |  |  |  | < 0.001 |
| No | 7267 (84.2) | 2031 (94.1) | 1931 (89.5) | 1784 (82.7) | 1521 (70.5) |  |
| Yes | 1365 (15.8) | 127 (5.9) | 227 (10.5) | 374 (17.3) | 637 (29.5) |  |
| Alpha_Blockers,n(%) |  |  |  |  |  | < 0.001 |
| No | 7319 (84.8) | 1959 (90.8) | 1888 (87.5) | 1812 (84) | 1660 (76.9) |  |
| Yes | 1313 (15.2) | 199 (9.2) | 270 (12.5) | 346 (16) | 498 (23.1) |  |
| Beta_Blockers,n(%) |  |  |  |  |  | < 0.001 |
| No | 3387 (39.2) | 1011 (46.8) | 851 (39.4) | 762 (35.3) | 763 (35.4) |  |
| Yes | 5245 (60.8) | 1147 (53.2) | 1307 (60.6) | 1396 (64.7) | 1395 (64.6) |  |
| Anticoagulants,n(%) |  |  |  |  |  | < 0.001 |
| No | 1734 (20.1) | 510 (23.6) | 464 (21.5) | 436 (20.2) | 324 (15) |  |
| Yes | 6898 (79.9) | 1648 (76.4) | 1694 (78.5) | 1722 (79.8) | 1834 (85) |  |
| CCB,n(%) |  |  |  |  |  | 0.002 |
| No | 5071 (58.7) | 1343 (62.2) | 1254 (58.1) | 1241 (57.5) | 1233 (57.1) |  |
| Yes | 3561 (41.3) | 815 (37.8) | 904 (41.9) | 917 (42.5) | 925 (42.9) |  |
| Statins,n(%) |  |  |  |  |  | 0.272 |
| No | 1152 (13.3) | 270 (12.5) | 285 (13.2) | 284 (13.2) | 313 (14.5) |  |
| Yes | 7480 (86.7) | 1888 (87.5) | 1873 (86.8) | 1874 (86.8) | 1845 (85.5) |  |
| ARB,n(%) |  |  |  |  |  | < 0.001 |
| No | 4712 (54.6) | 1293 (59.9) | 1117 (51.8) | 1106 (51.3) | 1196 (55.4) |  |
| Yes | 3920 (45.4) | 865 (40.1) | 1041 (48.2) | 1052 (48.7) | 962 (44.6) |  |
| Antiplatelets,n(%) |  |  |  |  |  | < 0.001 |
| No | 1986 (23.0) | 554 (25.7) | 473 (21.9) | 442 (20.5) | 517 (24) |  |
| Yes | 6646 (77.0) | 1604 (74.3) | 1685 (78.1) | 1716 (79.5) | 1641 (76) |  |
| ACEI,n(%) |  |  |  |  |  | 0.032 |
| No | 7304 (84.6) | 1852 (85.8) | 1849 (85.7) | 1796 (83.2) | 1807 (83.7) |  |
| Yes | 1328 (15.4) | 306 (14.2) | 309 (14.3) | 362 (16.8) | 351 (16.3) |  |
| Diuretics,n(%) |  |  |  |  |  | < 0.001 |
| No | 4482 (51.9) | 1594 (73.9) | 1239 (57.4) | 963 (44.6) | 686 (31.8) |  |
| Yes | 4150 (48.1) | 564 (26.1) | 919 (42.6) | 1195 (55.4) | 1472 (68.2) |  |
| Amiodarone,n(%) |  |  |  |  |  | < 0.001 |
| No | 8011 (92.8) | 2092 (96.9) | 2046 (94.8) | 2001 (92.7) | 1872 (86.7) |  |
| Yes | 621 ( 7.2) | 66 (3.1) | 112 (5.2) | 157 (7.3) | 286 (13.3) |  |
| Digoxin,n(%) |  |  |  |  |  | < 0.001 |
| No | 8069 (93.5) | 2090 (96.8) | 2038 (94.4) | 1996 (92.5) | 1945 (90.1) |  |
| Yes | 563 ( 6.5) | 68 (3.2) | 120 (5.6) | 162 (7.5) | 213 (9.9) |  |
| Nitrates,n(%) |  |  |  |  |  | 0.099 |
| No | 2221 (25.7) | 523 (24.2) | 542 (25.1) | 565 (26.2) | 591 (27.4) |  |
| Yes | 6411 (74.3) | 1635 (75.8) | 1616 (74.9) | 1593 (73.8) | 1567 (72.6) |  |
| Outcome,n(%) |  |  |  |  |  | < 0.001 |
| No | 7772 (90.0) | 2087 (96.7) | 2013 (93.3) | 1935 (89.7) | 1737 (80.5) |  |
| Yes | 860 (10.0) | 71 (3.3) | 145 (6.7) | 223 (10.3) | 421 (19.5) |  |
| CKM,n(%) |  |  |  |  |  | < 0.001 |
| 0-3 | 851 ( 9.9) | 131 (6.1) | 165 (7.6) | 211 (9.8) | 344 (15.9) |  |
| 4 | 7781 (90.1) | 2027 (93.9) | 1993 (92.4) | 1947 (90.2) | 1814 (84.1) |  |
