# Supplementary material for: The association between C-reactive protein-triglyceride glucose index and all-cause mortality in patients with cardiovascular-kidney-metabolic syndrome: a single-center retrospective cohort study
Source: Front Cardiovasc Med. 2026 Jul 8;13:1832873. doi: 10.3389/fcvm.2026.1832873 (PMC13388307; doi:10.3389/fcvm.2026.1832873)
Supplement: Supplementary file 3 [file Supplementaryfile3.docx]

Supplementary File：Table S3-A Subgroup analysis of CTI for all-cause mortality in patients with stage 0-3 CKM syndrome

| Subgroup | Variable | n.total | n.event(%) | adj.HR.95CI | adj.P.value | P.for.interaction |
| --- | --- | --- | --- | --- | --- | --- |
| Sex |  |  |  |  |  | 0.405 |
| Female | CTI | 222 | 33 (14.9) | 1.42 (0.65~3.11) | 0.374 |  |
| Male | CTI | 629 | 158 (25.1) | 1.72 (1.27~2.33) | <0.001 |  |
| Age |  |  |  |  |  | 0.688 |
| ＜60 | CTI | 332 | 79 (23.8) | 1.29 (0.84~1.98) | 0.244 |  |
| ≥60 | CTI | 519 | 112 (21.6) | 2.11 (1.47~3.01) | <0.001 |  |
| Hypertension |  |  |  |  |  | 0.015 |
| No | CTI | 288 | 60 (20.8) | 2.42 (1.39~4.24) | 0.002 |  |
| Yes | CTI | 563 | 131 (23.3) | 1.56 (1.13~2.17) | 0.008 |  |
| Diabetes |  |  |  |  |  | 0.005 |
| No | CTI | 521 | 97 (18.6) | 2.62 (1.72~4) | <0.001 |  |
| Yes | CTI | 330 | 94 (28.5) | 1.32 (0.9~1.94) | 0.16 |  |
| Renal_disfunction |  |  |  |  |  | <0.001 |
| No | CTI | 608 | 96 (15.8) | 2.48 (1.67~3.67) | <0.001 |  |
| Yes | CTI | 243 | 95 (39.1) | 1.19 (0.8~1.76) | 0.391 |  |
| Diuretics |  |  |  |  |  | 0.005 |
| No | CTI | 300 | 32 (10.7) | 2.86 (1.41~5.76) | 0.003 |  |
| Yes | CTI | 551 | 159 (28.9) | 1.58 (1.18~2.12) | 0.002 |  |
| Nitrates |  |  |  |  |  | 0.041 |
| No | CTI | 352 | 88 (25) | 2.51 (1.56~4.04) | <0.001 |  |
| Yes | CTI | 499 | 103 (20.6) | 1.49 (1.02~2.17) | 0.04 |  |

Supplementary File：Table S3-B Subgroup analysis of CTI for all-cause mortality in patients with stage 4 CKM syndrome

| Subgroup | Variable | n.total | n.event(%) | adj.HR.95CI | adj.P.value | P.for.interaction |
| --- | --- | --- | --- | --- | --- | --- |
| Sex |  |  |  |  |  | 0.327 |
| Female | CTI | 2487 | 222 (8.9) | 1.62 (1.25~2.1) | <0.001 |  |
| Male | CTI | 5294 | 447 (8.4) | 1.5 (1.26~1.79) | <0.001 |  |
| Age |  |  |  |  |  | 0.135 |
| ＜60 | CTI | 1825 | 37 (2) | 1.64 (0.83~3.22) | 0.153 |  |
| ≥60 | CTI | 5956 | 632 (10.6) | 1.51 (1.3~1.75) | <0.001 |  |
| Hypertension |  |  |  |  |  | 0.972 |
| No | CTI | 2426 | 158 (6.5) | 1.73 (1.25~2.39) | 0.001 |  |
| Yes | CTI | 5355 | 511 (9.5) | 1.46 (1.24~1.73) | <0.001 |  |
| Diabetes |  |  |  |  |  | 0.948 |
| No | CTI | 4588 | 335 (7.3) | 1.48 (1.19~1.82) | <0.001 |  |
| Yes | CTI | 3193 | 334 (10.5) | 1.52 (1.24~1.86) | <0.001 |  |
| Alpha Blockers |  |  |  |  |  | 0.002 |
| No | CTI | 6654 | 493 (7.4) | 1.6 (1.35~1.89) | <0.001 |  |
| Yes | CTI | 1127 | 176 (15.6) | 1.23 (0.93~1.62) | 0.149 |  |
| CCB |  |  |  |  |  | <0.001 |
| No | CTI | 4574 | 372 (8.1) | 1.82 (1.5~2.23) | <0.001 |  |
| Yes | CTI | 3207 | 297 (9.3) | 1.19 (0.96~1.48) | 0.114 |  |
| Statins |  |  |  |  |  | 0.09 |
| No | CTI | 950 | 134 (14.1) | 1.81 (1.31~2.5) | <0.001 |  |
| Yes | CTI | 6831 | 535 (7.8) | 1.5 (1.28~1.76) | <0.001 |  |
